# FDG PET/CT in cancer: comparison of actual use with literature-based recommendations

**DOI:** 10.1007/s00259-015-3217-0

**Published:** 2015-10-30

**Authors:** Henrik Petersen, Paw Christian Holdgaard, Poul Henning Madsen, Lene Meldgaard Knudsen, Dorte Gad, Anders Eggert Gravergaard, Max Rohde, Christian Godballe, Bodil Elisabeth Engelmann, Karsten Bech, Dorte Teilmann-Jørgensen, Ole Mogensen, Jens Karstoft, Jørgen Johansen, Janne Buck Christensen, Allan Johansen, Poul Flemming Høilund-Carlsen, on behalf of the PET/CT Task Force of the Region of Southern Denmark

**Affiliations:** Department of Nuclear Medicine, Odense University Hospital, Sdr. Boulevard 29, 5000 Odense C, Denmark; Department of Nuclear Medicine, Vejle Hospital, Vejle, Denmark; Department of Medicine, Vejle Hospital, Vejle, Denmark; Department of Haematological, Odense University Hospital, Odense, Denmark; Department of Plastic Surgery, Odense University Hospital, Odense, Denmark; Department of ORL Head & Neck Surgery, Odense University Hospital, Odense, Denmark; Department of Clinical Physiology and Nuclear Medicine, Næstved Sygehus, Næstved, Denmark; Organ Centre, Aabenraa Sygehus, Aabenraa, Denmark; Department of Gynaecoligcal and Obstetrics, Aabenraa Sygehus, Aabenraa, Denmark; Department of Gynaecoligcal and Obstetrics, Odense University Hospital, Odense, Denmark; Department of Radiology, Odense University Hospital, Odense, Denmark; Department of Oncology, Odense University Hospital, Odense, Denmark; Department of Quality and Research/HTA, Odense University Hospital, Odense, Denmark

**Keywords:** PET imaging, Rapid Evidence Assessment, Recommendation, Lung cancer, Malignant lymphoma, Malignant melanoma, Head and neck cancer, Colorectal cancer, Gynaecological cancer

## Abstract

**Purpose:**

The Region of Southern Denmark (RSD), covering 1.2 of Denmark’s 5.6 million inhabitants, established a task force to (1) retrieve literature evidence for the clinical use of positron emission tomography (PET)/CT and provide consequent recommendations and further to (2) compare the actual use of PET/CT in the RSD with these recommendations. This article summarizes the results.

**Methods:**

A Work Group appointed a professional Subgroup which made Clinician Groups conduct literature reviews on six selected cancers responsible for 5,768 (62.6 %) of 9,213 PET/CT scans in the RSD in 2012. Rapid Evidence Assessment was applied, using the methodology of systematic reviews with predefined limitations to search PubMed, Embase and the Cochrane Library for articles published in English/Danish/Swedish/Norwegian since 2002. PICO questions were defined, data recorded and quality appraised and rated with regard to strength and evidence level. Consequent recommendations for applications of PET/CT were established. The actual use of PET/CT was compared with these, where grades A and B indicated “established” and “useful” and grades C and D “potentially useful” and “non-recommendable” indications, respectively.

**Results:**

Of 11,729 citations, 1,729 were considered for review, and 204 were included. The evidence suggested usefulness of PET/CT in lung, lymphoma, melanoma, head and neck, and colorectal cancers, whereas evidence was sparse in gynaecological cancers. The agreement between actual use of PET/CT and literature-based recommendations was high in the first five mentioned cancers in that 96.2 % of scans were made for grade A or B indications versus only 22.2 % in gynaecological cancers.

**Conclusion:**

Evidence-based usefulness was reported in five of six selected cancers; evidence was sparse in the sixth, gynaecological cancers. Actual use of PET/CT agreed well with recommendations.

**Electronic supplementary material:**

The online version of this article (doi:10.1007/s00259-015-3217-0) contains supplementary material, which is available to authorized users.

## Introduction

**Fig. 1:**
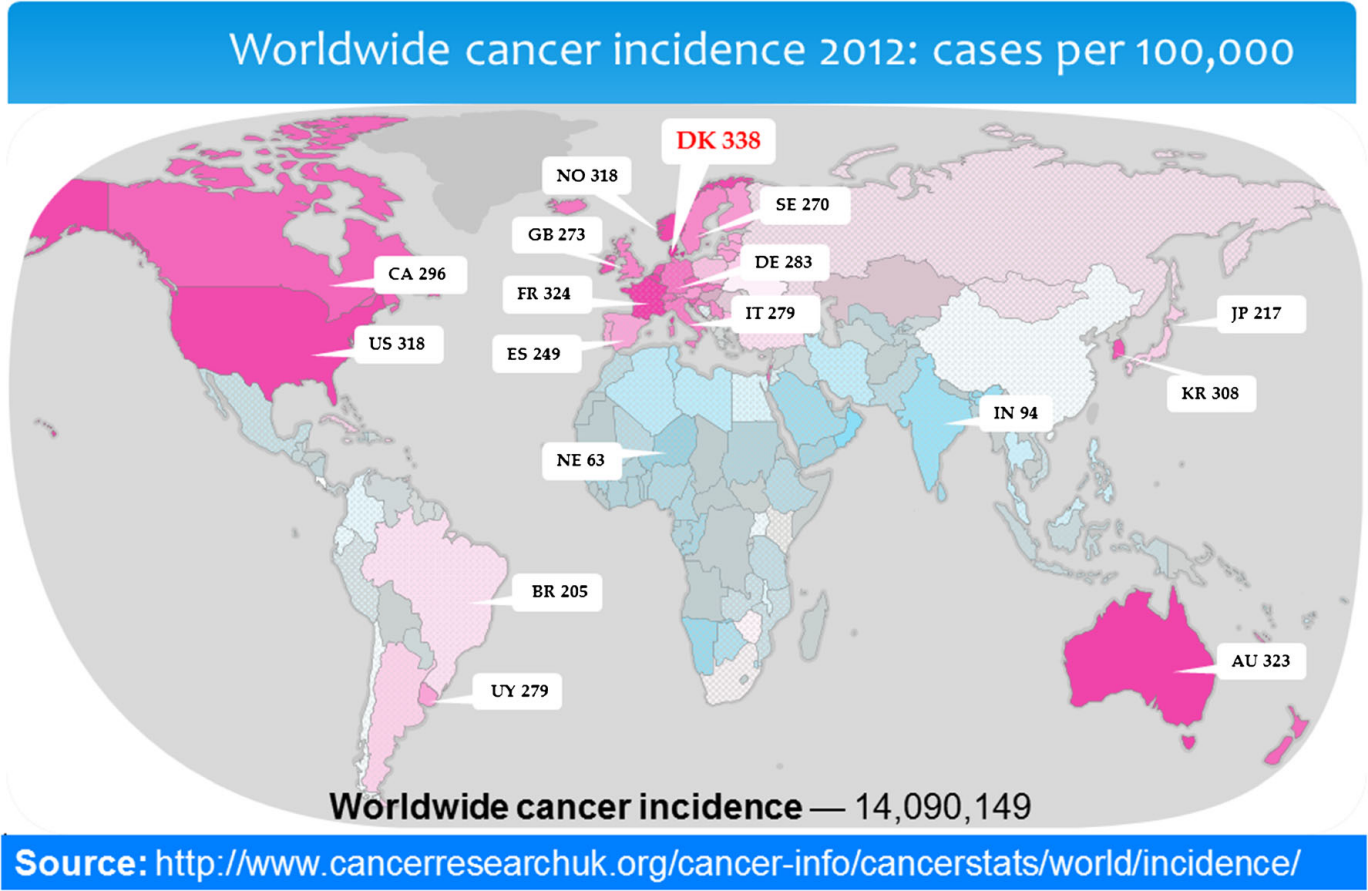
Worldwide cancer incidence, 2012 estimates. *Graded purple shades* indicate higher and *graded blue shades* indicate lower incidences, while *grey* indicates less reliable data; see http://globocan.iarc.fr. Accessed 7 Jul 2015

Health expenditure is on the rise again after the economic crisis and accounted for 9.3 % of the gross domestic product on average across Organisation for Economic Co-operation and Development (OECD) countries in 2012, well above 10 % in most Western European countries and much higher (16.2 %) in the USA [1]. Almost two thirds of OECD countries have experienced absolute decreases in pharmaceutical spending since 2009, whereas spending on hospital and outpatient care increased in many countries in 2012 [1], one reason being an increasing use of diagnostic tests including advanced diagnostic imaging [2]. Cancers figure among the leading causes of morbidity and mortality worldwide with 14.1 million new cancer cases, 8.2 million cancer deaths and 32.6 million people living with cancer (within 5 years of diagnosis) in 2012 worldwide, and the number of new cases is expected to rise by about 70 % over the next 2 decades [2, 3]. Corresponding numbers for Europe in 2012 were 3.7, 1.9 and 9.7 million and for the USA 1.6, 0.9 and 4.8 million, respectively [3–5]. Cancer alone constitutes a major source of expenditure due to expensive therapies and because multiple examinations are used for several purposes, i.e. diagnostic/staging, response evaluation, detection of recurrence and long-term follow-up [6–8]. In 2011 Denmark was assigned the dubious honour of being the world’s cancer capital with 226 new cases per 100,000 people [9], which rose to 338 new cases per 100,000 in 2012, compared to 273 in the UK, 284 in Germany, 296 in Canada and 318 in the USA (Fig. 1) [10]. These circumstances place healthcare decision-makers in a dilemma: Which diseases and treatments should be given priority? It is a tricky problem with many stakeholders, including patients, relatives, patient associations, medical specialties and healthcare personnel, not to mention pharmaceutical and medical technologies. What can be done?

**Fig. 2:**
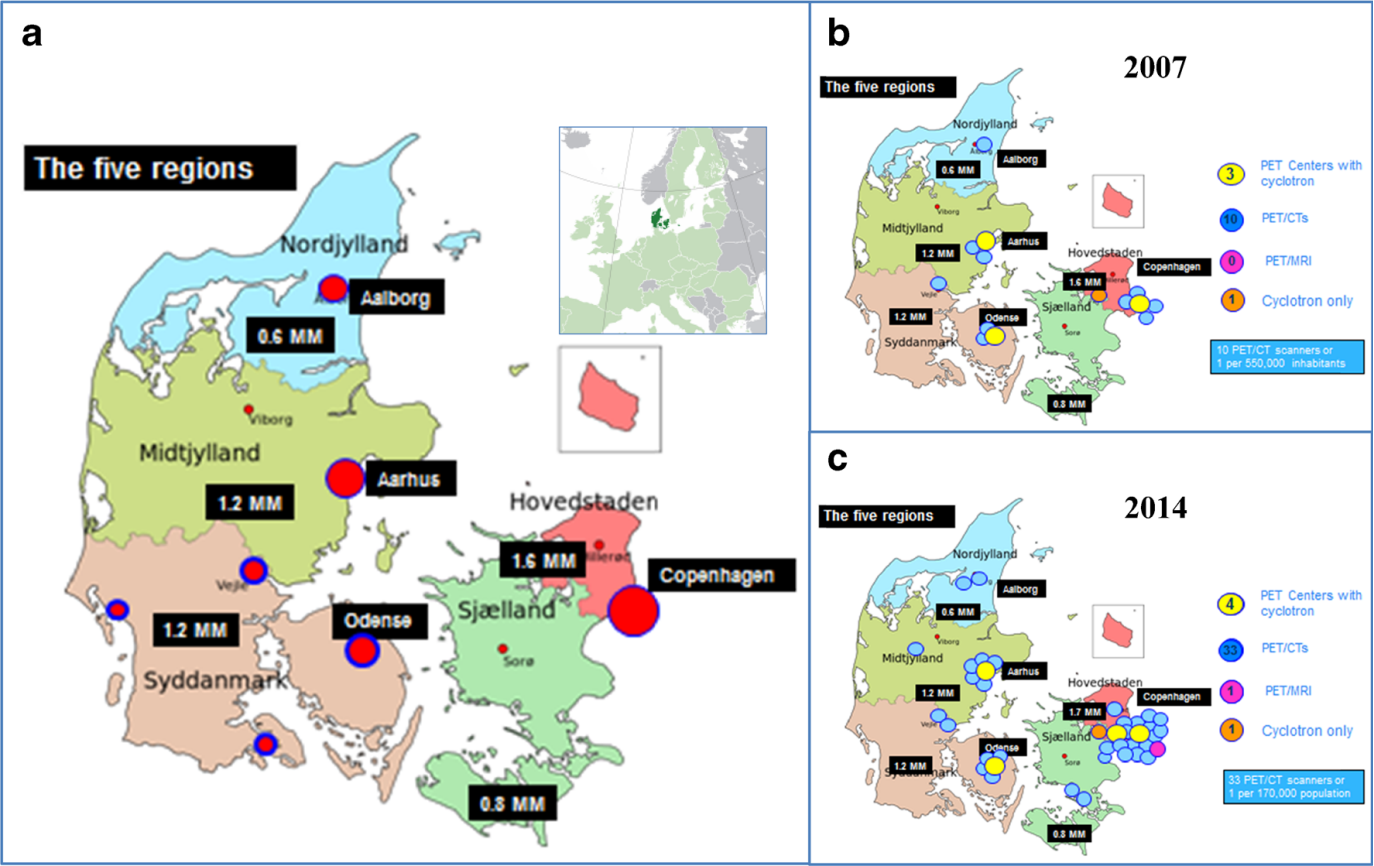
**a** The five regions of Denmark with a large insert (Denmark’s location in Europe) and a small insert (the Danish island of Bornholm located in the Baltic Sea). The RSD (*Syddanmark* on the map) connects Denmark with Germany (not shown) south of the Jutland Peninsula. The *red dots* surrounded by *blue circles* represent the four main hospitals of the region. **b** The number and placement of PET/CT scanners in Denmark in 2007. **c** The number and placement of PET/CT scanners in Denmark at the end of 2014

Numbers like these have created a massive demand for positron emission tomography (PET)/CT capacity in Denmark’s five administrative regions, including the Region of Southern Denmark (RSD) with its 1.2 of 5.6 million Danish inhabitants (Fig. 2). As a consequence, in 2013 the RSD appointed a Working Group, commissioned to work in two successive stages. In stage 1 to (1) identify and subdivide the evidence for the current use of PET/CT and give recommendations based on evidence strength and moreover to (2) compare the actual use of PET/CT in the RSD with established recommendations. Additionally to identify areas of future development and growth of PET/CT as input to stage 2, which aims to provide recommendations for a future strategy for the organization and use of PET/CT in the RSD. Stage 1 results were reported in April 2014 (available in Danish only at this link http://www.ouh.dk/wm442300) and form the basis of this article.

## Materials and methods

The work was organized at three levels: Working Group, Subgroup, and six Clinician Groups. The members of these groups are listed in the Appendix. The analysis should concentrate on clinically important patient-related outcomes in conjunction with relevant alternative examination modalities meaning that it should focus on studies in which PET/CT contributed to clinical endpoints like survival, change of treatment, etc. and exclude reports focusing exclusively on diagnostic accuracy. To ensure that the work could be completed within a reasonable time, it was decided to focus on six cancer areas, which by volume were the largest groups examined by PET/CT in 2012 at the two PET/CT centres in the RSD. The Working Group was chaired by the regional executive of healthcare in the RSD and comprised 19 further members representing four major hospitals of the region (Fig. 2). Each of these hospitals was represented by a hospital manager, head physicians and consultants in nuclear medicine, radiology and selected clinical specialties. Five members were assigned to a Subgroup comprising specialists in nuclear medicine, radiology and a clinical specialty, which together with a project coordinator was tasked to implement stage 1. For the literature review, the Subgroup suggested six Clinician Groups, one for each of six selected cancers that it was decided to focus upon, namely (1) lung, (2) (malignant) lymphoma, (3) (malignant) melanoma, (4) head and neck, (5) colorectal and (6) gynaecological cancer. The six Clinician Groups consisted typically of a contact person, i.e. a chief physician or professor of the relevant specialty, and a clinical consultant, who was exempted from ordinary service to perform the evidence review. Thus, the analysis of available evidence was carried out by trained clinicians, all of whom were users of PET/CT. The Subgroup supervised the work, compiled and prepared the final report.

### Literature review, level of evidence and consequent recommendations

Due to a short time line (terms of reference for the Working Group by 13 May 2013, first meeting 27 June, Subgroup tasks by 9 September, Clinician Groups established 1 November and final stage 1 report 19 May 2014), the literature review was undertaken as a Rapid Evidence Assessment (REA) and did not include references published after April 2014. REAs provide a balanced assessment of what is already known about an issue, “by using systematic review methods to search and critically appraise existing research. They aim to be rigorous and explicit in method and thus systematic but make concessions to the breadth or depth of the process by limiting particular aspects of the systematic review process” [11]. This was achieved by formulating specific questions guiding the review which need to be further limited if the question is broad; by using results of existing reviews to find primary studies; and by extracting primarily results and key data for simple quality assessment to conduct simple quality appraisal of studies. To ensure standardization, the searches were designed and carried out by the Medical Research Library of Odense University Hospital. They were confined to PubMed, Embase and the Cochrane Library, but were not limited as such. The extent of the review was adapted to each selected area. Two searches were carried out for each selected area: one for systematic reviews and meta-analyses only and another including all articles excluding only conference papers and books (Embase). Only articles in English, Danish, Swedish or Norwegian published since 2002 were included, since publications on PET/CT, in contrast to PET alone, were not available before then. Search terms (MeSH and free text) were suggested by the library and accepted by the Subgroup and Clinician Groups. A total of 53 search terms were used for PET/CT and the most common PET tracer, ^18^F-fluorodeoxyglucose (FDG), and additional 887 terms were used for the six cancer areas. Only studies about PET/CT (not PET/MRI or PET alone) were included, with the exception that some reviews and meta-analyses also included PET alone studies. The title and abstract of each study were reviewed by the relevant Clinician Group and studies focusing exclusively on diagnostic accuracy (sensitivity and specificity) were not included. In addition, criteria for outcome, comparator and possible patient courses were applied as determined by the Clinician Groups, which made additional limitations where relevant. In accordance with REA rules, the Clinical Groups were free to include/exclude based on the following criteria: (1) the methodological quality of the study being considered, (2) the relevance of that research design for answering the REA question and (3) the relevance of the study focus for answering the REA question. A filled out template defining PICO questions for each cancer area was used as guidance for the literature search and for sorting. PICO is a widely used critical appraisal tool and an acronym for population (e.g. patients with lung cancer), intervention (e.g. PET/CT), comparison (for the modality of comparison, e.g. CT alone) and outcome (e.g. relapse) [12].

Likewise, standardized evidence tables were filled out for each included article to record information on the area of research (e.g. lung cancer and described patient courses), source (authors, affiliations, journal, year of publication, etc., and number of references), objective, study design, study period, population and number of patients, results and comments.

Next, the evidence level of each article was rated according to a Danish version [13] of a standardized system devised by the Oxford Centre for Evidence-Based Medicine designating various levels of evidence to articles. At the time the project was commissioned, this methodology was the one endorsed by the Danish Health and Medicines Authority, in particular the “levels of evidence and grades of recommendations”. From January 2014 onwards the Danish authority recommends instead the GRADE system [14] for all national clinical guidelines. The methodology in our report follows the same transparent and structured approach as recommended by GRADE, only with adjustments regarding the breadth and depth of the literature. The highest levels (1a, 1b, 1c) denote systematic reviews/meta-analyses, randomized controlled trials and absolute effect studies, respectively, whereas the lowest level (5) indicates expert opinion without explicit critical appraisal, or based on physiology, bench research or “first principles” [15]. Thereafter, the evidence levels of all studies answering a distinct PICO question were used to provide graded recommendations for the use of PET/CT. Grade A recommendation was used for consistent level 1 studies, B for consistent level 2 or 3 studies or extrapolations from level 1 studies, C for level 4 studies or extrapolations from level 2 or 3 studies and D for level 5 evidence or troublingly inconsistent or inconclusive studies of any level. “Extrapolations” were where data were used in a situation that had potentially clinically important differences than the original study situation [15]. Finally, based on the literature review, level of evidence and recommendation grade, each Clinical Group prepared a summary with a conclusion and their recommendation for the use of PET/CT. This was filled into another standardized report form including also their estimates for further developments in the use of PET/CT within each particular area.

### PET/CT activity in the RSD and its agreement with recommendations

Using this material and colour codes for the potential clinical applications of PET/CT in cancer, i.e. diagnosis, staging, response evaluation, targeting of radiation therapy, disease control and recurrence detection, the Subgroup compared the results of the review and the Clinician Groups’ recommendations with the actual use of PET/CT in the RSD according to 2012 statistics. For this comparison, the three gynaecological cancers (ovary, cervical and uterine) were handled separately.

### Prediction of future development and growth

The report forms of the Clinician Groups including estimates for further developments in the use of PET/CT served as the basis for estimates prepared by the Subgroup for development and growth in PET/CT in years to come.

## Results

### Literature survey, level of evidence and consequent recommendations

Of 11,729 retrieved citations, 1,729 were considered for review, and 204 were included for the purposes of this survey, the summarized results of which are given in Supplementary Tables 1–6 in Online Resource 1. The amount and quality of articles within each cancer type varied considerably and was in general more comprehensive and of higher quality within lung, haematological and head and neck cancer than colorectal cancer, malignant melanoma and gynaecological cancers, where documentation was often heterogeneous, sporadic or lacking. The following was of note within the six cancer groups (for details see also Supplementary Tables 1–6 in Online Resource 1).

### Lung cancer

**Fig. 3:**
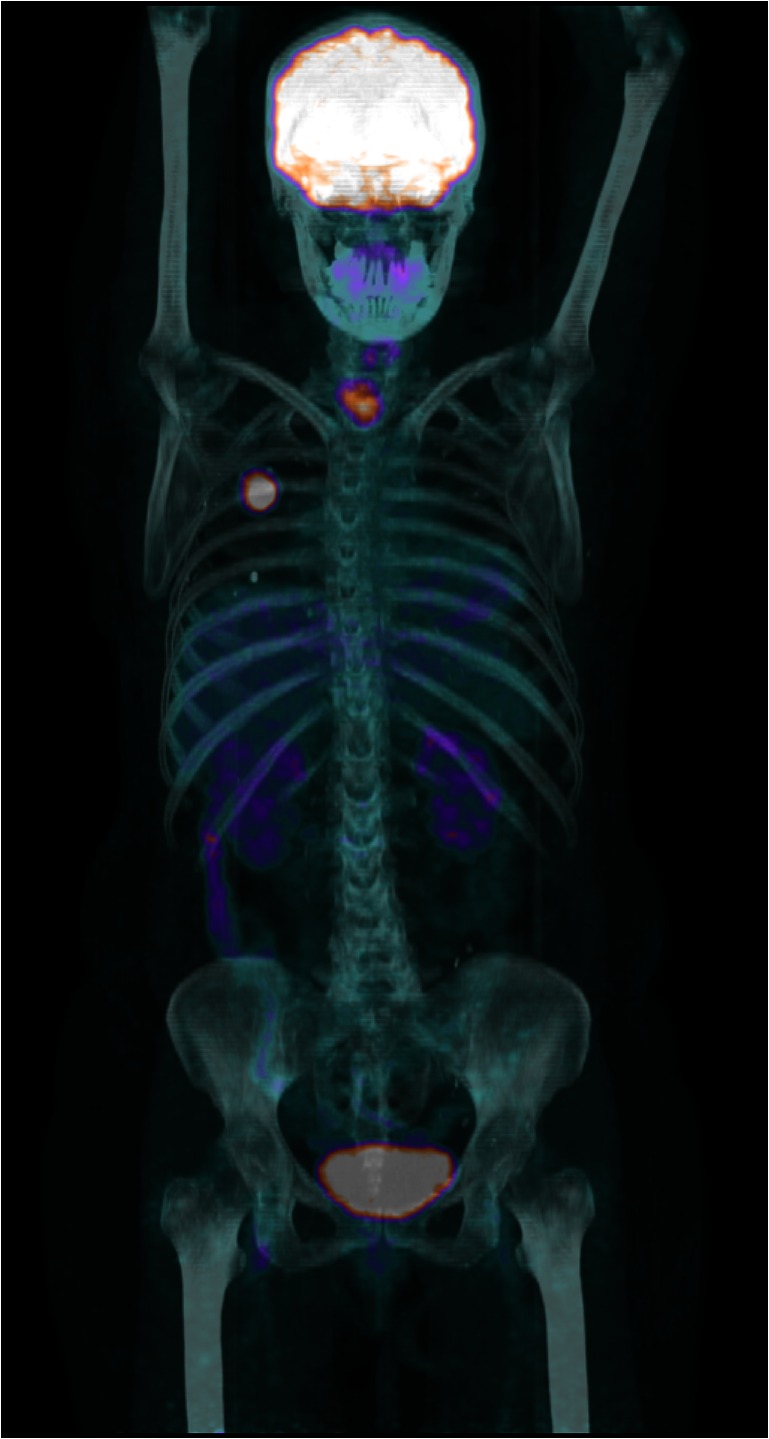
A scan showing one of the powers of PET/CT: In a patient with an isolated tumour in the upper right lobe of the lung, PET/CT revealed a potential lymph node metastasis just above the centre of the right diaphragmatic dome and an unknown lesion in the thyroid gland, not detected during work-up or by other imaging, but verified by biopsy following PET/CT

The use of PET/CT in the work-up of solitary pulmonary nodules may substantially reduce the number of invasive examinations [15–20] (Fig. 3). PET/CT prior to intended curative therapy may lower the number of futile operations by about 20 % [21]. A negative PET/CT in patients with suspected adrenal metastases may eliminate the need for biopsy [22].

### Malignant lymphoma

There was good evidence to recommend PET/CT for staging, response and end evaluation of patients with Hodgkin’s and diffuse large B-cell lymphoma [23–28]. Considerable beneficial effect has been reported in the shape of change in stage and therapy and with regard to prediction of progression-free survival [29, 30]. PET/CT helps to identify patients not in need of additional radiotherapy and is recommended before high-dose chemotherapy with stem cell support in patients with relapse, as all studies showed shorter progression-free survival with PET/CT positivity [31–34]. Thus, PET/CT may play a future role for the selection of patients for this kind of therapy.

### Malignant melanoma

It is recommended that all patients found to have metastatic malignant melanoma (stage 3) according to sentinel node diagnostics are offered additional staging with PET/CT, as this is the most sensitive way of diagnosing distant metastases [35–37]. When examining patients with high-risk metastatic melanoma (stages 3–4), there is literature consensus that PET/CT is the best modality to identify melanoma metastases [36–40]. PET/CT can detect unrecognized metastases, which may lead to altered management of 10–19 % of patients [40–43].

### Head and neck cancer

Studies indicate that PET/CT should be recommended in the diagnostic work-up and staging [44, 45]. In unknown primaries in the head and neck region, PET/CT identifies at least 30 % of primary tumours not detected by conventional means [46, 47]. PET/CT optimizes restaging of patients with recurrence [48, 49].

### Colorectal cancer

PET/CT is of benefit for the decision of surgery for liver metastases if CT or MRI is equivocal and/or to rule out distant metastases or secondary cancers obviating curative intervention [50, 51]. PET/CT can be of use in suspected recurrence, when CT is negative [52, 53], and PET/CT is recommended as first choice in patients with increased suspicion of recurrence [54, 55].

### Gynaecological cancers

PET/CT is recommended for response evaluation in cervical cancer, being superior for evaluation of treatment efficacy, and is a predictor of event-free and overall survival [56, 57]. On suspicion of advanced uterine cancer PET/CT is recommended for the choice between surgical or systemic treatment [58]. Studies in uterine cancer have low levels of evidence, but do show power of PET/CT for the detection of local relapse and distant metastases, as this may cause a change in management in 22–35 % of cases [59, 60]. PET/CT is recommended in patients with suspected recurrence of ovarian cancer due to elevated cancer antigen 125, but with negative CT and MRI [61]. Finally, PET/CT is considered the most accurate method for restaging from assessment of spread to the peritoneum, lymph nodes and local relapse [62, 63].

### PET/CT activity in the RSD and its agreement with recommendations

As shown by colour coding in Table 1, there was a clear and purposeful relationship in that the vast majority of PET/ CT scans were performed on indications that have good evidence (green and yellow markings), while only a small proportion was performed on indications of low or lacking evidence (orange and purple markings).

**Table 1:**
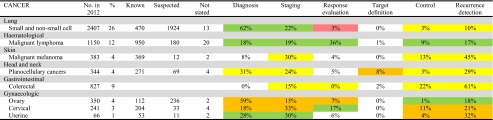
PET/CT scans in the RSD 2012

No. of PET/CT scans:

Total, survey 5768 Number of scans in the six selected cancer types (lung, haematological, skin, head and neck, gastrointestinal, gynaecologic)-

Total, in RDS 2012 9213 Total number of scans (the number above plus scans for other purposes)

Recommendations:

### Prediction of future development and growth

FDG will remain the dominant tracer, but new tracers are needed in the routine, for specialized functions, and for development of molecular imaging in general [64–67]. Solid-state PET detectors imply improved sensitivity, detection of smaller lesions and higher patient throughput [68]. PET/CT will be used in more cancers and often with more than one to two scans per patient [69], and PET/CT will play a role in infections of unknown origin [70], inflammation [71, 72], neurological disorders [67, 73–75] and cardiovascular disease, probably replacing myocardial scintigraphy [76–78].

## Discussion

### Principal findings

When comparing the actual use of PET/CT with the recommendations derived from the review there was a high agreement except in gynaecological cancer. Thus, when summarizing the percentages of scans actually used for each purpose in each type of cancer (see horizontal lines in Table 1), the sum of percentages that was in agreement with established (green colour code) or useful (yellow colour code) indications were (in rounded-off numbers) 97 % in lung, 99 % in lymphoma, 88 % in melanoma, 87 % in head and neck and 98 % in colorectal cancer. In these five cancers, only 3 % of scans (in lung patients) were with an indication of limited usefulness (purple colour code), whereas in 8 % (in head and neck patients) the indication was “potentially useful, albeit with minimal documentation” (orange colour code). The remaining 0–12 % of indications in each of these five cancers were not covered in this review. In gynaecological cancers, the picture was less uniform. In uterine cancer, 58 % of a low number of scans were used for an established indication (green colour code). In ovarian and cervical cancers, only 19 and 17 % of scans had an established indication (green colour code), whereas the remaining 81 and 83 %, respectively, were made for a “potentially useful indication, albeit with minimal documentation” (orange colour code). In gynaecological cancers, the remaining 0–6 % of scans were made for indications not covered in this review (Table 1). Continued development and growth of PET/CT was foreseen in all six cancer areas, and in additional cancer types, in infections and inflammation, and cardiovascular and neurological diseases.

### Strengths and weaknesses

A major strength of this review with recommendations was that it was undertaken by the clinical users and not experts in nuclear medicine. In theory, the latter category might have an interest in promoting PET/CT, while conversely recommendations provided by clinicians might underestimate the usefulness of PET/CT, the more so, the less the clinicians knew about PET/CT. Apparently, this type of bias was not prevalent as most clinical participants were experienced in the use of PET/CT, and because the recommendations were based on literature evidence and not on user experience.

Unfortunately, the review did not include all cancers and non-malignant diseases. To cover all potential applications of PET/CT was impossible if the review should have sufficient depth and quality within the limited time frame provided by the healthcare decision-makers. The chosen six cancer types accounted for almost two thirds of actually performed PET/CT scans in the RSD in 2012, i.e. 5,768 of a total of 9,213. We chose to use the method of REA allowing us to catch just about all existing literature within each area before predefined restrictions were used to reduce the number of articles to make the task manageable in due time.

### Other studies

We are not aware of similar reviews undertaken by the users. There are multiple studies on the accuracy of PET and PET/CT by specialists in radiology and/or nuclear medicine. The most comprehensive one was a tabulation of literature on FDG PET in oncology, cardiology and neurology published before July 2000 and prepared from documents submitted to the Health and Care Financing Administration (HCFA) in the USA [today’s Centers for Medicare and Medicaid Services (CMS)] in 2000 to request expanded Medicare reimbursement for FDG PET [79]. It showed an average estimated FDG PET sensitivity and specificity across all oncology applications at 84 % (based on 18,402 patient studies) and 88 % (based on 14,264 patient studies), respectively, and an average estimated management change across all applications of 30 % (based on 5,062 patient studies). The average accuracies for the six cancer areas of our review across all applications ranged then between 87 and 94 %, while the change in management averaged 25 % (range 5–37 %). This extensive type of survey was never repeated. The number of randomized trials on FDG PET/CT remains low [80], and emerging systematic reviews are inherently limited to answer only a single or a few key questions [81]. Two Danish studies, based on 743 PET and 6,056 PET/CT scans, reported a change in management with PET and PET/CT in 43 and 36 % of cases, respectively [82, 83].

Clinical PET came to life in 1976 [84], but its use remained experimental for a quarter of a century. However, when in 1999 the HCFA announced coverage for FDG PET in solitary lung nodules it was the starting signal of an almost explosive growth which was further boosted with the appearance of PET/CT scanners in 2001 and the CMS giving coverage in 2005 for a number of applications in several cancers, provided the cases were registered in a National Oncology PET Registry. Early reports from this registry estimated that PET/CT caused a change in management in about one third of cases [85, 86]. A major health technology assessment in the UK, based on literature up to August 2005 covering mainly stand-alone PET, aimed to assess the clinical effectiveness of FDG PET in breast, colorectal, head and neck, lung, lymphoma, melanoma, oesophageal and thyroid cancers [54]. Based on 6 systematic reviews and 158 primary studies, the strongest evidence of the clinical usefulness was found in lung cancer, for restaging Hodgkin’s lymphoma and staging/restaging colorectal cancer. It was concluded that clinical audit and further research was needed, but also that the PET clinical effectiveness could be extrapolated to cover PET/CT as the latter appeared to be slightly more accurate. Since then, two or more new generations of PET/CT scanners have reached the market and visual comparison alone can verify significant improvements. However, the literature on the clinical benefit of PET/CT is still limited. Randomized studies on clinical outcome are in short demand and should preferably focus on patient-relevant outcomes and cost-effectiveness [80, 81, 87]. Precise mapping of individual patient courses is required for documentation of clinical benefit. This is often not possible in the USA and Southern European countries because of frequent private clinics and practices. The situation is different in Northern Europe with countries like Denmark, because access to healthcare is free, the private sector is minimal and personal ID and nationwide databases make patient follow-up almost 100 % complete over time. The PET/CT infrastructure in Denmark is well established with rates of PET/CT scanners per million inhabitants exceeding the European average [42] and nuclear medicine centres within easy reach for most inhabitants. Therefore, multicentre studies from these countries may be the way to go to document the clinical effects of PET/CT and PET/MRI.

### Meaning of the study: possible explanations and implications for clinicians and policymakers

This review, other studies and market analyses foresee that PET/CT will continue to develop significantly for years to come in cancer and other diseases. The relative lack of documentation is not a phenomenon confined to PET/CT. It applies also to conventional imaging modalities, the use of which has been steadily increasing until recently [88, 89]. The molecular principle is unique and allows detection and monitoring of disease and its response to therapy often much earlier than what is possible with conventional imaging [67, 73, 90]. It is, therefore, not surprising that clinicians and healthcare policymakers are interested in this technology, its options and to what extent its use will benefit patients in an affordable way.

### Unanswered questions and future research

Do we need PET/CT or not? The concept of PET/CT is fundamentally different from that of traditional imaging with X-ray, CT or MRI. They display structure and allow for measurement of some physiological parameters. PET/CT can display and quantify molecular processes in the entire body in a single noninvasive examination with a molecular sensitivity much higher than with conventional modalities [91]. Problems with limited spatial resolution of PET were obviated and an increased diagnostic accuracy obtained with the introduction of PET/CT. However, diagnostic accuracy alone does not suffice unless consequent downstream changes in patient management are translated into improved patient outcome to a degree justifying purchase of additional scanners. The present literature reviews and the consequent recommendations were prepared by users rather than providers of PET/CT, which other things being equal may have a greater impact on administrators’ and policymakers’ decisions than those of imaging professionals. However, much still needs to be done to prevent PET/CT from becoming just another excellent examination instead of another’s preferred successor. The next step in our local process is to use this review to suggest strategies for the future organization and use of PET/CT in the RSD. These suggestions will be published elsewhere.

### Conclusion

This review suggested evidence-based usefulness in five of six selected cancer types and sparse documentation in the sixth type, gynaecological cancers. Actual clinical use of PET/CT agreed well with evidence-based recommendations. Development and growth was foreseen in many cancers and in other major types of disease. This calls for concomitant quality assessment strategies to ensure proper clinical implementation.

## Epilogue

This article is neither a typical original paper, nor a typical review. It is basically a summary of a commissioned work and its key results: The owners of the hospitals in the RSD wanted to know if there is sufficient evidence for the clinical use of PET/CT, and if its actual use in the RSD agrees with the available evidence. Users and not providers of PET/CT carried out the reviews and established the recommendations for comparison with the actual use of PET/CT in the region and, thus, their work and the comparison could not be influenced by radiologists and nuclear medicine specialists among the author team.

This type of report does not leave much room for additional reflections on the PET/CT method and its role in cancer management and, therefore, an epilogue may be an appropriate way to state a few professional views. FDG is the by far most common PET tracer being used worldwide in more than 90 % of cancers in clinical practice, based on a longstanding validation over the past few decades [67, 92]. PET/CT is changing the paradigm in cancer management from simple lesion measurement to lesion and whole-body characterization supporting a new era in personalized cancer therapy [93]. Despite an array of new PET tracers, the clinical recognition of these is slow in coming, and not only due to lengthy, cumbersome and expensive test procedures. For example, tracers characterizing cell proliferation, hypoxia or angiogenesis are scientifically highly interesting in providing new insight into disease mechanisms, but in the clinical setting they appear to be redundant to FDG, because the cumulative effects of the underlying biology are reflected by the levels of glucose metabolism, which is what is depicted by FDG PET (Kwee T, Gholami S, Werner TJ, Alavi A, Høilund-Carlsen PF. FDG, as a single imaging agent in assessing cancer, portrays the ongoing biological phenomena in many domains; do we need additional tracers for clinical purposes?; submitted). Studies should address challenges with using the standardized uptake values to discriminate malignant from benign lesions and follow changes over time during various therapeutic regimens. With novel quantitative techniques including partial volume correction and global disease assessment, FDG PET/CT has the potential to become an even more powerful modality in day-to-day practice of oncology. To demonstrate convincingly the clinical utility of PET/CT there is a dire need of large, multicentre and randomized studies, the number of which is surprisingly low [80]. In times of budget constraint, cost-effectiveness aspects of using PET/CT [87] should also be considered to ensure a balanced basis for decisions about the position of PET/CT in tomorrow’s healthcare.

### Electronic supplementary material

Online Resource 1 (DOCX 57 kb)

## Appendix

### Working Group members

RSD: Jens Elkjær (director of healthcare, chair), Frederik Frederiksen (head medical technology), Morten Jacobsen (secretary health staff). Sydvestjysk Sygehus: Bjarne Normark (medical director), Søren Steen Nielsen (head nuclear medicine), Niels Peter Rønnow Sand (consultant cardiology). Odense University Hospital: Peder Jest (medical director), Allan Johansen (head nuclear medicine), Henrik Petersen (consultant nuclear medicine), Jørgen Johansen (consultant, oncology), Jens Karstoft (head radiology), Ole Mogensen (professor, gynaecology-obstetrics), Nina Drøjdal Ryg/Janne Buck Christensen (HTA consultants, Dept. of Quality and Research/HTA). Sygehus Lillebælt: Niels Nørgaard Pedersen (head & director), Paw Holdgaard (consultant nuclear medicine), Bente Sørensen (head oncology). Sygehus Sønderjylland: Søren Aggestrup (medical director), Harro Bitterling (head radiology), Zofia Grzywacz (consultant oncology), Harald Flor (head neurology and circulation).

### Subgroup members

Odense University Hospital: Jens Karstoft (head radiology), Allan Johansen (head nuclear medicine), Henrik Petersen (consultant nuclear medicine), Ole Mogensen (professor, gynaecology-obstetrics), Nina Drøjdal Ryg/Janne Buck Christensen (HTA consultants, Dept. of Quality and Research/HTA). Sygehus Lillebælt: Paw Holdgaard (consultant nuclear medicine).

### Clinician Group members

Lung cancer: Ejler Ejlersen (head) and Poul Henning Madsen (consultant) (internal medicine, Vejle). Haematological cancer (lymphomas): Lene Meldgaard Knudsen (head haematology, Odense). Malignant melanoma: Morten Bischoff-Mikkelsen (head) and Dorte Gad and Anders Eggert Gravergaard (consultants) (plastic surgery, Odense). Head and neck cancer: Christian Godballe (professor) and Max Rohde (PhD student) (ear-nose-head surgery, Odense). Gynaecological cancers: Ole Mogensen (professor gynaecology and obstetrics, Odense) and Dorte Theilmann-Jørgensen (registrar anaesthesia, Vejle). Colorectal cancer: Karsten Bech (head organ center, Aabenraa), Bodil Engelmann (PhD student clinical physiology and nuclear medicine, Næstved).
