# Supplementary material for: FDG PET/CT in cancer: comparison of actual use with literature-based recommendations
Source: Eur J Nucl Med Mol Imaging. 2015 Oct 30;43:695–706. doi: 10.1007/s00259-015-3217-0 (PMC4764641; doi:10.1007/s00259-015-3217-0)
Supplement: Supplementary file 1 — (DOCX 57 kb) [file 259_2015_3217_MOESM1_ESM.docx]

**Online Resource 1 to**

***FDG-*PET/CT in cancer: comparison of actual use with literature-based recommendation.** By Petersen H, Holdgaard PC, Madsen pH, Knudsen LM, Gad D, Gravergaard DE, Rohde M, Godballe C, Engelmann BE, Bech K, Teilmann-Jørgensen D, Mogensen O, Karstoft J, Johansen J, Christensen JB, Johansen A, Høilund-Carlsen PF. **Eur J Nucl Med Mol Imaging.**

**Supplementary table 1** Summarized evidence: lung cancer

| **Lung cancer** | **Diagnosis** | **Staging** | **Staging** | **Control** |
| --- | --- | --- | --- | --- |
| PICO questions and  outcome measures | Can PET/CT reduce no. of invasive exams and complications without overlooking solitary lung nodules? | Can PET/CT reduce no. of superfluous intended curative therapies (surgery or radio-chemotherapy)? | Can PET/CT reduce no. of invasive exams for staging looking for mediastinal and distant metastases? | Can PET/CT detect recurrence earlier than CT and thus potentially improve management? |
| Advice | In non-high risk patients with a solid > 1 cm nodule, PET/CT can substantially reduce no. of invasive exams. | Routine PET/CT prior to intended curative therapy can reduce the no. of superfluous operations by about 20%. | (a) Better than CT, mediastinal biopsies still needed. (b) Limited evidence in liver metastases. (c) Adrenal biopsies not needed if negative PET/CT. | (a) Few studies, no clear pros and cons.  (b) Two retrospective studies suggest that PET/CT might be superior to CT. |
| Recommendation | A | A | A | B (maximal) |
| Evidence level | 1b | 1b | 1a-b | 2b |
| Reservations and comments | (a) Studies lacking in nodules < 1 cm. (b) PET positives need biopsy verification. | (a) Best effects in borderline operable/non-operable cases. (b) PET positives need verification. (c) PET/CT has unknown value when low risk of spread. | (a) PET/CT may reduce no. of invasive exams of mediastinum if low stage IA or in presence of obvious metastases. (b) Consequences of potential brain and bone mets not considered due to lack of biopsy proof. | (a) Lack of well-designed studies. (b) Existing studies mostly on value of PET/CT if CT is suspicious. (c) If PET/CT control turns out to be clinically useful, the demand for scans will raise considerably. |

**Supplementary table 2** Summarized evidence: hematological cancers

| **Hodgkin’s lymphoma** | **Diagnosis/staging** | **Response evaluation** | **End evaluation/control** | **Before high-dose chemotherapy** |
| --- | --- | --- | --- | --- |
| PICO questions and  outcome measures | Can PET/CT help to up and downstage and to change management? | Prognostic value of PET/CT at interim and final evaluation with regard to progression free survival (PFS)? | Can PET/CT diagnose relapse? | Has PET/CT prognostic value in relapse patients prior to the high dose chemotherapy with stem cell support? |
| Advice | PET/CT recommended for initial diagnosis/staging. Studies show altered stage (up to 41%) and treatment (up to 25%). More have ‘curative’ treatment, less therapy with heavy side effects. | PET/CT for mid evaluation: Increased PFS if negative PET except in one study only. End: Same trend. One study (1b): If negative PET, save radiotherapy of residual tumor on CT. | Poorer prognosis in PET positive than negative patients. Relapse in 3.9%-5%. Study too small and heterogeneous to recommend PET/CT for routine follow-up end of treatment. | PET/CT recommended prior to high dose chemotherapy. All studies showed significantly shorter PFS in PET/CT positive patients. |
| Recommendation | A/B | Middle A/B. End A | Insufficient literature | A/B |
| Evidence level | 1b and 2b | 1b and 2b | 1b and 2b | 1a and 2b |
| Reservations and comments | Significant clinical impact of PET-CT, but varying from study to study. | Mid-term PET/CT has clinical impact. Ongoing studies with PET/CT-guided management. | Only 4 studies. | Significant clinical impact of PET-CT, but varying from study to study. |
| **Diffuse B-cell lymphoma** |  |  |  |  |
| PICO questions and  outcome measures | Can PET/CT help to up and downstage and to change management? | Has PET/CT prognostic value at mid-term with regard to PFS? | Has PET/CT prognostic value and can it diagnose relapse? | Has PET/CT prognostic value in relapse patients prior to the high dose chemotherapy with stem cell support? |
| Advice | PET/CT recommended for initial diagnosis/staging. Studies show altered stage (up to 42%) and treatment (up to 45%). More have ‘curative’ treatment, less therapy with heavy side effects. | PET/CT recommended for mid and final evaluation. Mid: 13/17 studies show increased PFS by negative PET. | PET/CT recommended for end evaluation after chemotherapy to find out who would benefit from radiotherapy of residual tumor. Not recommended for routine control to detect relapse. | PET/CT recommended prior to high dose chemo-therapy. All studies showed significantly shorter PFS in PET/CT positive patients. |
| Recommendation | A/B | A/B | End A. Control D | A/B |
| Evidence level | 1b and 2b | 1b and 2b | 1b and 2b | 1a and 2b |
| Reservations and comments | Significant clinical impact of PET-CT, but varying from study to study | Mid-term PET/CT has clinical impact. Ongoing studies with PET/CT-guided management. | Varying evidence. End evaluation by PET/CT is part of international response criteria; response to primary therapy is an indicator in Denmark. | It is expected that future studies will use PET/CT prior to high-dose chemotherapy with stem cell support as guidance for therapy. |
| **Follicular lymphoma** |  |  |  |  |
| PICO questions and  outcome measures | Can PET/CT help to up and downstage and to change management? |  | Has PET/CT prognostic value and can it diagnose relapse? |  |
| Advice | Follicular lymphomas may transform into highly malignant variant in certain regions. This may not always be  diagnosed by biopsy or CT. Instead PET/CT is recommended. |  | End: PET positivity post treatment means shorter PFS.  Control: PET/CT not recommended except in suspected high-malignant. |  |
| Recommendation | D |  |  |  |
| Evidence level | 2b |  | End 1b+2B. Control 1b |  |
| Reservations and comments | Four retrospective studies: PET/CT causes upstaging in 18%-40% and  changed treatment in 18% of cases. |  |  |  |

**Supplementary table 3** Summarized evidence: malignant melanoma

| **Malignant melanoma** | **Staging** | **Staging** | **Control** | **Relapse** |
| --- | --- | --- | --- | --- |
| PICO questions and  outcome measures | Effect of PET/CT compared to patient-related outcome parameters? | Effect of PET/CT compared to patient-related outcome parameters? | Effect of PET/CT on management and outcome? | Effect of PET/CT on therapy in patients with relapse? |
| Advice | Stage I and II patients (no metastases found at primary work-up) have no benefit of PET/CT. Sentinel node technique is golden standard for distinguishing between these and patients with metastases (stages III and IV). | Patients with metastasizing disease (stages III and IV) should be offered PET/CT for therapy triage. | In stage III and IV PET/CT is best modality for identifying melanoma metastases and good at detecting unknown metastases, which may cause change of management in 10%-19% of these patients. | Level II evidence for the usefulness of PET/CT for therapy triage in patients with clinical relapse in that PET/CT may confirm or change the operative plan or point to chemotherapy instead. |
| Recommendation | B | B |  |  |
| Evidence level | 3b | 3b | 3b | II |
| Reservations and comments | It is recommended that Stage I and II patients are managed according to the protocol of the Danish Melanoma Group, but specialists may defer from this if indicated. | According to the literature PET/CT is superior in diagnosing distant melanoma metastases, except metastases to liver and brain. | No solid literature evidence that the use of PET/CT will increase survival, but indications that management change due to PET/CT might.  In stage I and II patients PET/CT may have a role to play in ulcerated melanomas. |  |

**Supplementary table 4** Summarized evidence: head and neck cancer

| **Head and neck cancer** | **Diagnosis/staging of squamous cell carcinoma** | **Diagnosis of unknown primary tumor** | **Control/relapse of squamous cell carcinoma** |
| --- | --- | --- | --- |
| PICO questions and  outcome measures | Has PET/CT superior diagnostic and staging accuracy compared to conventional imaging? | Has PET/CT superior diagnostic and staging accuracy compared to conventional imaging in patients with cancer of unknown primary (CUP**) in the head and neck? | Is PET/CT more useful than conventional imaging for the detection of recurrence? |
| Advice | PET/CT seems to have better diagnostic accuracy, particularly with respect to spread of malignancy.  PET-CT provides better staging and, through that optimized therapy planning. Similarly, studies indicating altered clinical management due to PET/CT.  PET/CT is therefore recommended for diagnostic work-up and staging. Studies do not provide information on effect on survival. | PET/CT has better accuracy with regard to detection of the primary tumor than conventional diagnostic imaging. Detects minimum 30% primary tumors not be shown conventionally. PET/CT is therefore recommended for diagnostic work-up and staging. Better classification may optimize treatment planning significantly. No information about effects on survival and limited about effects of PET-induced changes of treatment plan. | PET/CT seems to have better diagnostic accuracy, particularly with respect to spread of malignancy.  This means better re-staging and significantly optimized treatment planning. Therefore PET/CT is recommended in suspected clinical recurrence.  PET-CT may have a place in standardized control and may render a multiannual control program superfluous. The evidence for this is too sparse to give rise to recommendations. |
| Recommendation* | B | B | B |
| Evidence level | 2a | 2a | 2a |
| Reservations and comments | A major ongoing Danish study aims to uncover how much up-front PET/CT improves treatment planning. The clinicians conducting the evidence survey in head and neck cancer find that PET/CT provides accurate and clear information of the patients’ cancer disease, especially the degree of spread. It is expected that PET/CT will become the most important examination in the work-up of head and neck cancer. | Conclusions are consistent with guidelines of the National Danish Head and Neck Cancer Group (DAHANCA). Based on the literature survey and the surveyors’ clinical experience PET/CT is considered the most important examination in the diagnosis of CUP. Concrete evidence for clinical outcome measures will probably be available in a foreseeable future. | The ongoing Danish study aim also to uncover the clinical value of PET/CT in suspected recurrence and to what degree PET/CT can replace conventional examinations and make the course of patients with relapse faster, more simple and possibly cheaper. Based on information retrieved through the present literature survey and the surveyors’ clinical experience it is expected that PET/CT will become the most important examination in the diagnosis of relapse. |

* Recommendations modified in keeping with the Oxford Centre for Evidence-Based Medicine Levels of Evidence and Grades of Recommendations.

** Addendum to CUP: The results were reinforced by two Danish national prospective studies showing that the use of PET or PET-CT had clinical implications in 25% of patients due to recognized primary cancer, metastatic disease, or detection of secondary cancers in the body using PET-CT.

**Supplementary table 5** Summarized evidence: colorectal cancer

| **Colorectal cancer** | **Staging** | **Staging** | **Response evaluation** | **Control** | **Relapse** |
| --- | --- | --- | --- | --- | --- |
| PICO questions and  outcome measures | Can PET/CT substitute CT in primary staging? | Can PET/CT provide more accurate preoperative assessment before resection of liver and lung metastases? | Can PET/CT predict efficacy of chemo-radiotherapy and identify inefficient therapy earlier? | Is PET/CT of use in routine postoperative control of radically operated patients? | Is PET/CT of use in suspected relapse, thus causing change of management? |
| Advice | Too few and too small studies available to judge. | Some indication of benefit of PET/CT for judgment of operability of distant metastases. | Studies with promising results, but too heterogeneous and no randomized to allow judgment. | Insufficient evidence. Apparently of use in selected patients with suspected recurrence. | PET/CT maybe of use if symptoms or increased CEA suggest relapse and CT is negative. First choice if heightened suspicion. |
| Recommendation | B | B | B | B | B |
| Evidence level | 2a-2b | 1b-2b | 2a | 1b-2a | 2a |
| Reservations and comments | CT is the primary exam, but PET/CT is supplementary (and used in 5-6% of cases) if suspicion of metastases. | Studies exclusively on liver and not lung metastases.  PET/CT might have role for control after metastasectomy. | Particularly interesting for downstaging to facilitate operation of metastases or tumor and discard ineffective therapy. | No consensus on the use of PET/CT for this purpose.  Danish departments offer CT besides endoscopy and clinical follow-up. | High risk of relapse following metastasectomy, but CT assessment is difficult, why PET/CT might have a place. |

This table is mainly based on articles with clinical patient-relevant primary endpoints. Articles focusing on the diagnostic value of PET/CT in terms of sensitivity and specificity and comparison with other diagnostic methods were not considered.

**Supplementary table 6** Summarized evidence: gynecologic cancers

| **Cervical cancer** | **Diagnosis/staging** | **Response evaluation** | **Control/relapse** |
| --- | --- | --- | --- |
| PICO questions and  outcome measures | Has PET/CT clinical impact? | What is the effect of PET/CT? | What is the effect of PET/CT? |
| Advice | Insufficient literature evidence to advocate PET/CT in all patients with suspected cervical cancer. Strong suggestion that PET/CT should be used for diagnosis/staging whenever advanced disease is suspected. PET/CT may predict prognosis prior to treatment. | Post therapy response by PET/CT is a predictor of event-free and overall survival. PET/CT is superior for early evaluation of response compared to ultrasound, CT, MRI. Pre-therapy PET/CT advocated in high-risk patients; may optimize brachytherapy. | PET/CT advocated in suspected relapse if raised biomarkers and inconclusive conv. imaging. Weak studies suggest PET/CT post curative therapy to identify need of further treatment. Economic benefit of PET/CT unproven in weak economic model. |
| Recommendation | C | A | C |
| Evidence level | 1-4 | 1 | 1-4 |
| Reservations and comments | Suboptimal for early staging. Advocated in advanced disease. Superior (compared to CT and MRI) with regard to lymph node metastases. | Studies report change/modification of therapy in 15%, 27% and 40% of cases due to PET/CT. | Few studies with few patients. PET/CT is considered the most accurate imaging modality in various types of relapse. PET/CT can predict relapse. |
| **Uterine cancer** |  |  |  |
| PICO questions and  outcome measures | Effect of PET/CT versus conventional assessment? |  | Effect of PET/CT versus conventional assessment? |
| Advice | PET/CT not recommended in initial work-up, except if suspicion of advanced disease with extra-pelvic spread. PET/CT may optimize surgical treatment by identifying metastases or suggest systemic therapy. PET/CT is superior in evaluation of lymphadenopathy (> 5 mm) indicating a worse prognosis. |  | Too little strong evidence to suggest general control by PET/CT. PET/CT has high accuracy in detection of local recurrence and distant metastases with/without symptoms, causing management change in 22%-35% of cases. Clinical impact is not documented, could be of great value, though. |
| Recommendation | A | N/A | C |
| Evidence level | 1 and 4 | N/A | 4 |
| Reservations and comments | As of now, PET/CT cannot substitute N staging by peroperative lymphadenectomy. |  | Negative PET/CT suggests better prognosis. Control does not influence prognosis or relapse diagnosis. |
| **Ovarian cancer** |  |  |  |
| PICO questions and  outcome measures | What value for the patient can PET/CT provide? | Can PET/CT assess response to chemotherapy? | Effects of PET/CT compared to conventional assessment? |
| Advice | Too little evidence to generalize. However, consensus to advocate PET/CT preoperatively for correct staging ensuring optimal treatment. Primary PET/CT allows assessment of interventions and gives indications for further treatment. PET7CT should be offered in stage IV disease. PET7CT provides additional information compared to tvUS, MRI and CT. | Too little evidence to generalize. All studies have a positive attitude to monitoring with PET/CT  during/after chemotherapy to counteract treatment failure/optimize therapy/avoid side effects. PET/CT is considered best bet for image evaluation of chemotherapy response compared to tvUS, CT, and MRI due to earlier detection of treatment failure. | PET/CT is recommended in suspected recurrence based on rise in CA-125 and negative CT/MRI, as PET/CT plus CA-125 have greater overall accuracy. PET/CT may be the most accurate imaging modality for restaging when spread to peritoneum, lymph nodes and local recurrence. PET/CT suggests management change in 25%-60% of cases. |
| Recommendation | C | C | A |
| Evidence level | 4 | 4 | 1 |
| Reservations and comments | Uncertainty about handling occasional findings. PET/CT can change prognosis of individual patients and groups due to "stage migration". | Only 6 review articles available reviewing 8-16 single studies, all of evidence level 4. | Due to problems with small (<5-7 mm) lesions, PET/CT cannot substitute endoscopic re-staging. Use of PET/CT may cause better quality of life. |
